# The Principles of Preventive Inoculation and Serum Therapeutics, with Considerations Concerning Their Practical Application1An address delivered before the Section for Pathology of the Academy of Medicine of Buffalo, N. Y., January 21, 1896.

**Published:** 1896-03

**Authors:** A. C. Abbott

**Affiliations:** Philadelphhia, First Assistant, Laboratory of Hygiene, University of Pennsylvania


### BUFFALO MEDICAL JOURNAL.

VOL. XXXV. MARCH, 1896. No. 8.

### Original Communications.

### THE PRINCIPLES OF PREVENTIVE INOCULATION AND SERUM THERAPEUTICS, WITH CONSIDERATIONS CONCERNING THEIR PRACTICAL APPLICATION.[1]

By A. C. ABBOTT, M. D., Philadelphhia,

First Assistant, Laboratory of Hygiene, University of Pennsylvania.

THE topic on which I have the honor to address you this evening is one that has, by reason of its importance, claimed a great deal of attention from scientific medical men during the past few years.

When it was suggested that I take as the text of my remarks the practical outlook of the results of recent investigations along the lines of protective vaccination and serum therapeutics, I acquiesced with pleasure, as this theme would give us an opportunity of discussing together the outcome of work the underlying principles of which are, I believe, destined to ultimately place the prevention and treatment of disease on a higher scientific plane than they have ever before occupied. In making this prediction, I am fully aware of the risk that is taken,—a risk that may indeed appear to be unjustifiable when viewed in the light of the limited number of instances in which the possibilities of the successful employment, in human beings, of preventive vaccination and serum therapeutics have been demonstrated. But, while these successful demonstrations have been few in number, we must keep before us the fact that their underlying principles have rested upon a foundation firmly grounded in unimpeachable experimental proof, and are capable, we believe, of further elaboration and wider application.

For a period extending over more than the first half of the present century the single instance of an effort to imitate a method

1. An address delivered before the Section for Pathology of the Academy of Medicine of Buffalo, N. Y., January 21, 1896.

of nature in affording immunity to infectious maladies was the practice of protective inoculation and vaccination against small-pox. Prior to the introduction of vaccination by Jenner it had been observed that after recovery from a mild attack of an affection known as cow-pox, that simulates in certain of its details, though considerably modified in degree, the disease small-pox, the individuals so affected were henceforth, in most instances, proof against the ravages of small-pox. It had also been noticed, and is still a constant observation, that a single nonfatal attack of certain forms of infection often leaves the patient proof against subsequent inroads of the same malady. It was observations of this character that induced the query respecting the cause of the phenomena, and though the riddle is not as yet by any means solved, still, during the past decade and a half, so much light has been shed upon the manifold sides of the question through accurate, laborious, fundamental research, that we are at present tolerably sanguine as to the ultimate outcome.

In the early days of studies in this field, the ideas that were advanced in explanation of immunity and susceptibility belonged very largely to the realm of the hypothetical. They were without foundation in truth and when tried in the balance of experimental test were found to be sadly wanting. From the time of Jenner's demonstration, in 1798, to the effect that it was possible to protect human beings against small-pox by vaccination with the lymph obtained from the cow-pox vesicle, a phenomenon of which we know little more now than he did then, up to 1880, nothing was contributed to our knowledge of the subject. In 1880, in the course of his studies upon the cholera of chickens, Pasteur showed, for the first time, that by artificial means it was possible to so modify the virulence of the bacteria causing this disease that they would no longer produce fatal results, but, instead, only temporary local disturbances, and that in these cases the chickens that had recovered from such a modified attack were not now susceptible to the inroads of the highly virulent bacteria that cause the fatal form of the infection.

In view of the fact that at about the same date (1878–'81) Pasteur, Koch, Toussaint and others had shown the rod-shaped organisms discovered by Pollender (1855) and by Davaine (1863) in the blood of animals dead of anthrax to be the etiological factors concerned in this disease, it is not surprising to find the observations of Pasteur utilised in a scheme for the production of a vaccine against this much dreaded disease. Without entering into technical details, it will suffice to say that as a result of numerous trials by different experimenters, it was ultimately demonstrated that by the employment of various agencies, thermal and chemical, drying and prolonged cultivation under particular artificial circumstances, it was possible to do to the virulent anthrax bacillus just what Pasteur had done to the bacillus of chicken cholera—namely, to so attenuate its virulence that it no longer killed susceptible animals, but caused instead only temporary disturbances from which the animals recovered. With recovery they were usually found to be no longer susceptible to the more severe, commonly fatal, form of the infection. We can justly say that it was in the course of these studies on anthrax that the foundation stones were laid for our knowledge of protective vaccination with attenuated living virus.

Of fundamental importance to our understanding of the processes of immunity is the fact that the constitutional symptoms and pathological lesions of disease are the results of the toxic activities of metabolic products of the bacteria concerned in the production of disease, and that immunity, as well as disease, is established by these substances, not alone when elaborated within the tissues of the animal to which the bacteria have gained access, but also when they are produced under artificial conditions and purposely introduced into susceptible animals.

An advance of great importance was made in our knowledge of immunity through the application of this fact by Salmon and Smith.[2] They demonstrated that a certain sort of immunity to particular forms of infection might be conferred upon animals by injecting into them the filtered, germ-free products of growth of certain bacteria to the pathogenic influences of which the species of animal under treatment was highly susceptible.

This demonstration of the possibility of inducing immunity through purely chemical, or biologico-chemical, means shed an entirely new light on the subject, though its true significance was not at the time, apparently, either understood or appreciated. As a result of numerous investigations suggested by this discovery of Salmon and Smith, immunity is today held by a number of those who have had most to do with the elaboration of our knowledge of it as a purely chemical phenomenon, a phenomenon that involves not the microörganisms themselves that are concerned in the production of disease, but rather the agencies through which they produce it—namely, their poisons.

As a result of very recent investigations this does not seem to be a safe generalisation, for evidence has been produced that leads us to believe that in particular cases the protecting substances in the body of the immunified animal possess in fact a much more conspicuous germicidal than antitoxic activity, and at times these two peculiarities appear to be in a measure coincident. (See 9 and 15 of bibliography.)

With the preceding observations as a base of operation, it is not surprising that many scientific men yielded to the temptation to investigate the manifold problems presented by this fascinating and at the same time almost unexplored field.

With the advent of modern bacteriological methods and their application to the study of the various forms of infection, our knowledge of the causation of these diseases rapidly became enriched. As in the majority of cases the factors concerned in the production of infection were shown to be bacterial in their nature, efforts were from time to time made to determine if the particular species of bacteria that had been isolated from particular forms of disease, and reasonably shown to stand in causal relation to them, could be made to respond to such treatment as had been employed in the preparation of vaccines against anthrax and against chicken cholera ; or if the products of growth of these bacteria were potent to afford immunity to animals into which they, devoid of the bacteria by which they had been formed, were injected. Neither failure nor success was the uniform outcome of these experiments. From every aspect, however, this field of work gave promise of being most fruitful, and no thought of discouragement was entertained ; indeed, the interest in the subject grew with tremendous strides, and among those who were and still are foremost with investigations in this particular line and its correlated branches may be numbered many of the most brilliant scientific minds of the day.

Without attempting to bring to your notice the experimental details of the work that has brought our knowledge of immunity to the point it now occupies, or to elucidate many of the interesting collateral phases of the subject, I shall occupy the time that remains with a consideration of those features that will, I believe, serve as the most striking illustrations of the lines along which the work has developed and which may also be taken as indices of the direction in which these studies will subsequently be pursued.

As a result of numerous observations we now know that immunity may be (1) natural, *i. e.*, certain races, certain species, certain individuals may from birth be wholly or in part insusceptible to forms of infection to which other races, species or individuals may fall victim. (2) Immunity may be acquired through natural channels, if such a term may be applied to the contraction of and recovery from disease as observed in the ordinary course of life. The acquisition of immunity through this means is, as you know, always more or less uncertain. To a number of those infected in the ordinary way the disease proves fatal; a certain number of the remainder are subject to recurrence of the disease, while a fair proportion are seen to have acquired a condition of body through which they are protected against subsequent attacks. (3) Immunity may be artificially produced, viz., by purposely inducing a very mild form of the disease by inoculation with vaccines that have been made by depriving the virulent bacteria of a portion of their pathogenic power; also by inoculation with infinitesimally small amounts of fully virulent virus, a plan that often results in the production of only a temporary constitutional disturbance that may be sufficient, however, to subsequently protect the animal under treatment; also by the induction of a condition of tolerance to the poisons of specific, virulent bacteria, either the poisons produced by the bacteria as products of their nutrition when grown under artificial circumstances, or the poisons obtained by particular methods from the protoplasmic bodies of the bacteria themselves, or both together. In this plan the germ-free poisons in their full degree of toxicity may be employed after having been highly diluted, or they may be modified by particular chemical or thermal treatment until robbed of much of their original potency. Lastly under this head, immunity may be conferred upon an animal by the injection into it of the serum of the blood of another animal previously immunified against the disease, and immunity may also be conferred to the nursing young through the milk of an immunified mother.

As a rule, in the artificial production of immunity, no sharp lines are drawn between the several preceding methods of work, but more often the plan adopted partakes of the nature of two or more of these schemes combined. Against a given disease several methods of inducing immunity may often be successful.

When by either of the foregoing plans an animal is robbed of its susceptibility to a particular form of disease, it is undoubtedly a most impressive result; but this is, to those engaged in the purely scientific study of the subject, of far less interest than the intimate nature of the phenomenon, the "why and the wherefore"

of it, so to speak. It is this curiosity of science that has induced so many brilliant medical minds of modern times to endeavor to solve this complicated problem, and it is through these efforts that our general knowledge of important vital functions of the animal economy has been greatly extended and enriched.

With the opening of this hitherto closed book by Pasteur and his pupils, it is not surprising that the importance and true meaning of its first pages should have been misinterpreted, and that hypotheses should have been advanced in explanation of the phenomena that were not sufficiently grounded in experimental proof to withstand the test of further investigation. Thus, for instance, Pasteur[3] expressed the opinion that immunity seen to follow a non-fatal attack of infection was due to abstraction from the tissues of materials necessary to the growth of the bacteria that caused the infection, and in this way the individual no longer offered conditions capable of supporting the life of this particular species of bacteria. Chauveau[4] believed acquired immunity to be due to the deposition within the body, during the primary attack, of substances that are detrimental to the growth of the species of bacteria concerned in that attack should they subsequently gain access. In the light of modern experiment these speculations have little more than historic interest.

Of a great deal more than historic interest, however, were the observations of Metchnikoff[5] (1884) that induced him to advance his theory of phagocytosis, in explanation of the intimate nature of the vital phenomena that are in operation during the efforts of the body to resist the invasion of pathogenic microörganisms. This most fascinating hypothesis was based upon the fact that certain wandering cells of the animal economy possess the property of taking up and digesting within their protoplasmic body many foreign particles with which they come in contact. It was the belief of Metchnikoff that bacteria were in this way taken up and digested by these wandering cells, and that in the performance of this function the amoeboid cells served as actual sentries and scavengers of the body. He believed that by the processes of inducing immunity the efficiency of the weapons of defense possessed by the wandering cells was increased to a point at which they were enabled to overcome and destroy disease-producing bacteria to which they would otherwise have fallen victim.

A severe blow was given to this idea by the elaborators of the humoral doctrine of immunity. Through the investigations of v. Fodor,[6] Nuttall,[7] Behring[8] and many others, the important fact was demonstrated that certain fluids of the body, especially the serum of the blood, are capable of destroying large numbers of bacteria, even when deprived of their cellular elements. It was demonstrated that this was not a property peculiar to the blood of naturally or artificially immunified animals, but was possessed to a varying degree, toward particular species of bacteria, by the blood of practically all animals. Contrary to what might have been anticipated, it was found that when drawn from the body the blood of certain animals that are markedly susceptible to particular forms of infection possessed a relatively high degree of germicidal activity toward the bacteria causing such infections ; whereas, on the other hand, the serum of the blood of certain other animals that are more or less naturally immune to these infections may be comparatively feeble in germicidal properties. In a single conspicuous instance it has been possible to reasonably refer the condition of natural immunity of an animal to the composition of its blood, but this represents an exceptional observation that is not generally applicable. I refer to the studies of Behring[8] upon the immunity of the rat to anthrax, where the natural immunity of the animal to. this disease is attributed to the high degree of alkalinity of its blood. In other instances the germicidal properties of the serum have been seen to increase after the establishment of a condition of artificial immunity ([9] and [15]).

Thus, step by step, the field was gradually contracted, and the question of prime importance now became that concerning the element or elements in the blood to which this remarkable germicidal activity could be attributed.

The details of the manifold chemical studies that were at once undertaken upon the blood, the tissues and other fluids and secretions of the body hardly come within the scope of a paper of this character. It will suffice to say that it was through studies projected along these lines that discoveries were made that are destined to be of the greatest importance in the elucidation of the subject.

Among the first of the important discoveries that were made upon the composition of the blood as regards its germicidal function was that of Buchner.[10] He demonstrated that the power possessed by the serum of the blood of rendering bacteria inert was a property peculiar to a living protoplasm dissolved in the serum, and that this body could be robbed of its germicidal properties by depriving it of certain of its salts by dyalisis, by excessive dilution, by moderately high temperatures and by other detrimental influences. For this or these germicidal proteids Buchner suggests the name "alexines."

It was subsequently demonstrated that if the poisonous products of growth of certain pathogenic bacteria be introduced into the body of a susceptible animal in nonfatal doses, or in a condition of diminished toxicity, that the effect of such treatment is exhibited by a more or less pronounced constitutional reaction on the part of the animal. After recovery from this temporary disturbance, the animal is often found to be not only insusceptible to infection by the bacteria by which the poison was manufactured, but the serum of its blood in certain cases has undergone a demonstrable change: it has acquired the property of neutralising the fully virulent poisons, though its property of destroying the bacteria themselves may not in all cases have been conspicuously altered. In other words, in the process of acquiring immunity the chemical composition of the blood is modified ; it is enriched by the addition to it, through changes in the body, of a substance that is antidotal to the poisonous products of the pathogenic bacteria against which the animal is immunified, without its relation to the bacteria themselves having been in all cases materially changed.

In this connection, it is important to note that it is possible by the repeated injections of nonfatal but gradually increasing doses of toxins into susceptible animals to finally increase the antitoxic value of the blood of that animal to a degree far in excess of that ever seen to exist in immunity acquired through an ordinary attack of disease, or the immunity that is induced simply as a prevention against bacterial invasion. It is in this way that antitoxic serums are obtained that are of sufficient strength, that is, contain sufficient amount of the antidote to be of service in the treatment of disease already in progress, a condition necessitating the neutralisation of large amounts of poison circulating in the body as speedily as possible with the greatest amount of antidote concentrated in the smallest bulk of the curative agent.

In the course of earlier investigations upon the subject, Buchner[11] offered the suggestion that the immunity conferred by a single attack of disease exists by reason of certain "*reactive changes*" that occur in the tissues during the disease, and that with the establishment of this alteration the animal acquires insusceptibility to further attacks of the same malady. Though much has been done on the subject since this hypothesis was advanced, we are today but little nearer the actual solution of the problem than that which is embodied in this view.

The opinion now generally held is that the tissues acquire, during the constitutional reaction coincident with the primary attack of the disease the property of generating the antidotal substance, though it is also believed, especially by Buchner, that the antidotal or antitoxic body is in some cases the poisonous products themselves of the bacteria so modified through the reaction of the tissues that they now possess protective, neutralising or antitoxic peculiarities. On these points, however, it is as yet impossible to speak with certainty.

The observation that the serum of the blood of a susceptible animal could be rendered antidotal to certain bacterial poisons by the gradual introduction into the animal of the poisons until a condition of tolerance was reached, together with the discovery that a certain group of highly pathogenic bacteria produce their effects, almost if not entirely, through poisons that they produce within the system, while they themselves are localised to some particular point within or upon the body, suggested a line of experiments having for their object the practical application of these observations to the treatment of disease resulting from the activities of what may be termed the truly *toxic* pathogenic bacteria. It was in the course of these investigations that Behring and Kitasato[12] made the important discovery that the serum of the blood of animals rendered tolerant to certain bacterial toxins not only afforded protection to these animals against the poisonous effects of these substances through antidotal properties, but that by the transference of serum from this immunified animal to another susceptible animal, that immunity was at once conferred upon the animal into which such serum had been injected. The original observation was made in the course of studies upon tetanus.

It was not long, however, before the principles upon which this observation rested were applied to the study of other forms of toxic infection with, as you know, the result of placing in our hands, through the labors of Behring and his associates, an agent whose favorable influence upon the course of the diphtheritic infection is seen to be so pronounced as to justify the opinion that with the introduction of the antitoxic serum in the treatment of diphtheria, an epoch has been marked in the history of medicine.

By some the method of inducing and transferring immunity, as elaborated by Behring and his colleagues, is considered as only the induction of a condition of tolerance to chemical poisons, *i. e.*, the rendering of an animal poison-proof (Giftfest), and not as a protection to bacterial infection. There is evidence, however, to indicate that this view is erroneous, and that the method is applicable in certain cases of true infection that are not characterised by marked toxic features.[13]

In the present state of our knowledge, it is impossible to say to what extent acquired immunity in human beings is due to the presence of antitoxic substances in the circulating fluids, or to indicate in how far the observations that have been made upon tetanus and diphtheria are applicable to other infections ; certainly, in so far as the truly toxic infections are concerned, one is constrained to feel sanguine as to the ultimate outcome of the further application of the principles on which the antitoxic method of treatment is based. As a precautionary measure, however, it may not be amiss to emphasise the impropriety of generalising from these single instances. We must bear in mind that the conclusions reached with regard to tetanus in animals, and diphtheria in man, are the results of observations having an incontestable experimental basis, without which any pseudo-scientific structure that we may rear through analogical reasoning will, sooner or later, totter and fall without a moment's warning. The wider application of these principles to the treatment of disease is only to succeed through the establishment of a firm basis of experimental proof for each separate and distinct affection. From this it is clear to those of you who are familiar with laboratory methods that there are many obstacles to be overcome, some of them, in our present position, almost insurmountable. The impossibility of faithfully reproducing in animals that we use for experiment some of the most important diseases to which human beings are liable, may serve as an example of one of the gravest of these difficulties.

From what has preceded, we observe that we must distinguish between three principal methods of inducing immunity—namely, by the activities of living bacteria in the tissues ; through the introduction into the body of the germ-free, poisonous products of bacteria ; and through the introduction into susceptible animals of the serum of the blood (and of other secretions) from another animal already immunified.

By either the first or second of these procedures the condition of immunity is established only after the lapse of the time necessary for the elaboration of the immunifying substances within the tissues; whereas, by the last method these substances that have already been prepared in the immuned animal from which the serum is obtained are transferred directly, and the animal receiving them is at once protected; as Ehrlich[14] conceives it, one simply transfers the protecting agent from one animal to another.

There is a further distinction as regards the results of these methods of procedure. The immunity that is induced through vaccination with attenuated virus, or conferred by the gradual introduction of toxins to the point of tolerance, simulates more closely in the degree of its permanence the immunity usually conferred by a nonfatal attack of infection contracted in the ordinary walks of life than does that produced by the injection of the serum of immunified animals.

Ehrlich[14] proposes to designate the more or less permanent immunity frequently conferred by an attack of an infectious disease as "active immunity," while for the immunity that is established through the direct transference of the immunising agent from the blood of one animal to the tissues of another he employs the name "passive immunity." This designation is not acceptable to all writers on the subject, the objection being that there is not as yet sufficient proof that the induction of "passive immunity" is as simple a matter as Ehrlich conceives it to be. There is some evidence in support of the idea that the real immunifying agent may not be contained in the immunifying serum, but that this serum is only instrumental in inducing the peculiar tissue reaction that results in the formation of the actual protecting body.

Another point in connection with this subject, on which there has been considerable controversy, is that concerning the specificity of the relation between the immunity inducing toxins and the antitoxic substances elaborated in the body as a protection against them. By the majority of investigators there is believed to be a specific antagonism between the poisons produced by a given infectious microörganism and the protective agent that is present in the body of the animal artificially immunified against this particular microörganism. Objections have been raised to accepting this as a law, on the grounds that the serum of artificially immunified animals is sometimes seen to possess protective properties, to a limited extent, against forms of infection or intoxication other than that against which the animal has been immunified. In this connection, it must be remembered that the *normal* serum of man, of horses, and occasionally of other animals, has also at times been observed to possess similar "*general*" antitoxic peculiarities. It may be that the observations on which are based the objections to the idea of a specific relation between particular toxins and their antitoxins can be explained through this normally present, universal, so to speak, antidote.

In a number of experiments, antitoxic properties of the serum against specific bacterial poisons have been induced through the induction of tolerance to the poisons of bacteria of a different species. This condition appears, however, to be little more than an accentuation of the normally present protective agent already referred to. It has never been possible to bring about in this manner as high or as permanent a degree of immunity against a particular disease as that which can be obtained by the use of the microörganism causing the disease, or the products of its growth.

Of fundamental importance in their bearing upon this subject are the remarkable observations of Pfeiffer.[15] He showed that it was easily possible to confer upon guinea-pigs a condition of immunity to Asiatic cholera by the repeated injections into them of sterilised cultures of the organism causing the disease. If upon the establishment of immunity he now injected into the peritoneal cavity of these animals an amount of the living culture that would otherwise certainly prove fatal, not only had this no effect, but within a few minutes, almost instantly, there was an actual disintegration of the organisms injected that could readily be followed with the microscope. He demonstrated, further, that this relation between the immune animal and the organisms against which it was protected was a specific one, and that no such disintegration occurred when other bacteria were injected. If, with the cholera spirillum other bacteria were injected, *only* the cholera spirillum was thus broken up. He showed, in addition, that while the serum of the blood of the immune animal was capable of conferring immunity to Asiatic cholera upon other animals not immune, it had no disintegrating effect upon the cholera spirillum when in contact with it in the test-tube, but if he injected into the peritoneal cavity of a nonimmunified guinea-pig the fatal dose of living cholera spirillum, and followed this immediately by an intraperitoneal injection of the serum from an immune animal, *at once* the disintegration of the bacteria within the peritoneal cavity was to be detected.

It will be seen that these investigations are of importance, not alone as regards the question of specificity, but also as regards the nature and origin of the protecting body, for we have here a serum from an immune animal capable of conferring immunity; capable, when injected into the susceptible animal, of endowing it with the peculiar germicidal function noted in the immune animal from which the serum originated, but still, totally incapable of this remarkable bactericidal activity when tested outside the animal body. Manifestly the real protective agent is generated by the tissues as a result of the specific irritation of a something contained in this serum.

We must remember, however, that our knowledge on this subject does not as yet admit of the laying down of hard and fast laws, and it is not unlikely that much of what we consider as sound today may tomorrow prove to be untrustworthy. We are in many respects hardly more than on the threshold of this many-sided subject.

Equal in interest and importance to any of the other problems relating to the question of acquired immunity, is that concerning its transmissibility from parent to offspring. Can the condition of immunity to particular infections and intoxications be inherited?

While there have arisen from time to time examples that serve to indicate the possibility of this question being answered in the affirmative, we are indebted to Ehrlich[14] for the experimental demonstration of the accuracy of these indications. In the course of a series of studies upon the vegetable toxalbumens, abrin, ricin and robin, especially as regards their intoxicating effects upon animals, and the methods of inducing immunity to them, he demonstrated the possibility of easily inducing in white mice, normally markedly susceptible to these poisons, a condition of resistance that enabled them to withstand large multiples of the otherwise fatal dose. He likewise conclusively demonstrated that females on whom such immunity had been conferred transmitted, through the milk, to their nursing young, an antitoxic substance that induced in them a condition of body through which they, too, were enabled to resist the otherwise fatal dose of the particular poison against which the mother was immunified. This transmission of immunity appears to be entirely a maternal function, the father, in Ehrlich's experiments, playing no part in the process.

From the preceding considerations of the subject, we see that a condition of immunity against certain forms of infection may be more or less easily acquired, and that when once acquired is to a varying degree transmissible to the offspring of the individuals who have experienced this modification.

In the light of these established facts, one might be tempted to consider the natural immunity seen to be possessed by certain individuals and races to particular forms of disease as, after all, an acquired trait,—acquired not as a result of the purposeful inoculation of progenitors with modified virus or attenuated toxins, but rather acquired through the processes of survival and hereditary transmission. For instance, one might argue: when a given number of individuals become affected with the same form of infection, those that survive are manifestly not only less susceptible to its inroads than were those that succumbed, but, as we have seen, the degree of this insusceptibility is further increased by the attack of the disease through which they have safely passed. These survivors, it might be claimed, transmit to their offspring not only certain mental and structural characteristics, but physiological peculiarities as well, among which may be a condition of insusceptibility to this particular form of infection that has been accentuated at the nursing period through the protecting influences of the milk of an immune mother. Still, in support of this view one might continue: the constant presence in a community of a certain form of disease is ultimately accompanied by a diminution of its virulence and a lower degree of fatality from it than is seen to follow its first or only occasional appearance, and that continuous exposure, therefore, of large numbers of individuals to particular diseases may result, through the natural phenomena of survival and inheritance, in developing a race endowed with natural insusceptibility to this malady.

Plausible and attractive as this view may appear on superficial examination, there are objections to its adoption.

In the strict sense of the word, and in the light of present knowledge, we must regard natural immunity as a trait that has been transmitted, and is further transmissible, through generations by parents in whom it is blastogenic. It is congenital, therefore, and inherent to the integral protoplasm of the individual or species endowed with it. There is no evidence of its having been acquired through any of the channels that apply to the acquisition of immunity. Its transmission, like other physiological peculiarities, is probably as much under the paternal as the maternal influence, and is lasting; whereas, the transmission of acquired immunity is a function only of the mother, and so far as we know, is of but temporary duration.

There can be no doubt that the constant exposure of a raçe of individuals to a disease is ultimately accompanied by a diminution of susceptibility of many of the individuals to this disease, but we have no evidence that this condition of increased resistance persists, and we cannot regard such tissue resistance as a genuine natural immunity.

To admit that the condition of natural immunity, in the sense in which the term is here used, represents, after all, the inheritance of an induced peculiarity, is to admit in general the possibility of the hereditary transmission of acquired traits, "an assumption that has often been made, but never yet proved."*

Natural immunity must as yet be considered as a vital property, inherent to the idioplasm, the intimate nature and workings of which cannot be explained. It distingushes the individual endowed with it only by its protective influences during exposure to particular forms of disease. It is not explainable through any demonstrable excess of protective characteristics of the body-fluids or tissues, contrary to what may usually be done in the case of artificially immunified animals, for, as stated above, the fluids of the body of the naturally immune animal may be neither more nor less germicidal or antitoxic than are similar fluids from animals that are naturally susceptible.

Manifestly the prevention and treatment of disease along the lines suggested by the investigations cited in this paper in many respects closely simulate some of the methods of Nature. It is from this standpoint that we believe the further elaboration and wider application of the principles involved in the processes of preventive inoculation, and serum therapeutics are destined to be of inestimable service in the advancement of the medicine of the future.

Already as a result of these labors, animals have been rendered more or less insusceptible to a number of different infections and intoxications, for instance, to chicken cholera, anthrax, erysipelas, symptomatic anthrax, malignant edema, hog cholera, typhoid fever, hemorrhagic septicemia, vibrionic septicemia, Asiatic cholera, diphtheria, tetanus, pneumococcus infection, pyocyanus infection,

---

* *Weismann*—Essays on Heredity and Kindred Biological Problems ; Essay on Retrogressive Development in Nature, page 14, Vol. II. Edited by Poulton and Shipley Oxford, Clarendon Press. 1892.

proteus infection, infection or intoxication by bacillus coli communis, and infections by pyogenic cocci.

Not only has the possibility of conferring immunity to these infections been demonstrated, but in the case of certain of them the serum of the blood of the immunified animals has been found to possess properties that can be utilised in the treatment of these infections or intoxications after they are already in progress in other animals. Thus, for example, the treatment of diphtheria by the antitoxin method comes under this head. In the case of erysipelas, the experiments of Marmorek[16] and others indicate similar possibilities. Marmorek found in the blood of rabbits immunified against infection by the streptococcus of erysipelas, a substance that he states possesses curative powers over the disease when it is already in progress in nonimmunified animals. Pfeiffer and Kolle[17] have detected in the blood of animals rendered tolerant to the typhoid toxin a substance that is germicidal to the typhoid baccillus, and Beumer and Peiper[18] find a nongermicidal, but rather an antitoxic substance in the blood of animals artificially immunified to the typhoid poison. Beumer and Peiper state that this serum possesses not only immunising powers, but that the poisonous effects of the typhoid toxins can be neutralised by the subsequent injection of the antitoxic serum. They believe, therefore, that the serum possesses curative virtues. Yersin, Borel and Calmette[19], in their studies upon bubonic plague, obtained from the blood of animals rendered immune to this infection an actively antitoxic serum that they hope to ultimately utilise in the treatment of the disease. The studies on vaccinia lead to the belief that there exists in the blood of the vaccinated animal a substance possessing certain antagonistic relations to the active principle of vaccine lymph[20], whatever that may be. In this case, as with scarlatina, both experiments and results are unsatisfactory, because of the important unknown factors that come into play. From recent investigations it seems probable that the treatment of tetanus by means of antitoxic serum, as is shown to be possible in animals, will, before a very great while, be successfully extended to the disease in men. The treatment of Asiatic cholera by the antitoxin method is apparently destined to be an outcome of the very near future, and finally, the experiments bearing upon the treatment of tuberculosis by means of antitoxic substances is predicted by Behring to be soon successfully demonstrated. Behring and Knorr already claim to have detected in the blood of animals rendered tolerant to the poisonous influences of tuberculine, (the toxin produced by the bacillus tuberculosis,) a body, antituberculin, that possesses the property of robbing tuberculin of its poisonous peculiarities.[21]

When we contemplate this array of practical results and bear in mind that they are the outgrowth of experiments made with a definite purpose, each step of which was directed toward a particular object, and that through these experiments susceptible animals have been, and may at will be, rendered more or less immune to a large number of diseases of bacterial origin, there is justification for the statement "that the problems relating to immunity and infection have been, in part at least, removed from the realm of pure hypothesis and placed in a position favorable to exact experimental solution."[22]

LITERATURE REFERRED TO IN THIS PAPER.

1. *Pasteur*—Comptes rendus, Acad. des Sci., Tome xci., 1880, p. 673.

2. *Salmon & Smith*—Proc. Biolog. Soc., Washington, D. C., Vol. III., 1886: Cent. f. Bact u. Parasitenkunde, Bd. 2, 1887; Trans. IX. Internat. Med. Congress, Washington, 1887.

3. *Pasteur*—Bull. d. l'Acad. d. Med., 1880.

4. *Chauveau*—Comptes rendus, d. l'Acad. d. Sci., 1880, No. 91.

5. *Metchnikoff*—Arb. a. d. Zoologischen Inst. d. Univ. Wien, 1884, Bd. V.; Fortschritte p. Med., 1884, Bd. 2: Virchow's Archiv., Bd. 96, p. 177, and Bd. 97, p. 502; Annales d. l'Inst. Pasteur, 1887, Tome I., p. 321.

6. *v. Fodor*—Deutsche med. Woch., 1887, No. 34; Cent. f. Bact. u. Parasitenkunde, 1890, p. 753; Ebenda, 1895, p. 225.

7. *Nuttall*—Zeit f. Hygiene, Bd. IV., 1888, p. 353.

8. *Behring*—Cent. f. Klin. Med., 1888, No. 38; Die Bekampfung der Infektionskrankheiten, Leipzig, 1894.

9. ——— *and Nissen*—Zeit. f. Hyg., 1890, p. 412.

10 *Buchner*—Arch. f. Hyg., 1890, Bd. X., p. 149–173.

11. *Buchner*—Eine neue Theorie über Erzielung von Immunität gegen Infektionskrankheiten, Muenchen, 1883

12. *Behring & Kitasato*—Deutsche med. Woch., 1890, Bd. XVI., p. 1113.

13. Consult *Kitt*—Cent. f. Bact u. Parasitenkunde, 1893, p 869; *Lorenz*—Ebenda, 1893, p. 357, and 1894, p. 278.

14. *Ehrlich*—Deutsche med. Woch., 1891, Nos. 32–44. Zeit. f. Hyg. u. Infektionskrankheiten, 1892, Bd. XII., p. 183; ——— *and Hübener*. Ebenda, 1894, Bd. XVIII., p. 51.

15. *Pfeiffer*—Zeit. f. Hyg. u. Infektionskrankheiten, Bd. XVIII., p. 1; Ebenda—Bd. XX., p. 198.

16. *Marmorek*—La semaine med., 1895, No. 17, Annales d. l'Inst Pasteur, 1895, Tome IX.; consult also *Roger*—Ebenda

17. *Pfeiffer & Kolle*—Deutsche med. Woch., 1894, No. 48.

18. *Beumer & Peiper*—Zeit. f. klin. Med., Bd. XXVIII., Heft 3 and 4

19. *Yersin, Calmette & Borel*—Annales d. l'Inst. Pasteur, 1895, No. 7.

20. For literature on this subject consult *Sternberg & Reed*, Transactions Associations of American Phys., 1895, Vol. X, p. 57.

21. *Behring*—Address delivered before the 67th meeting of Naturalists, at Lübeck—Deutsche med. Woch., 1895, No. 38.

22. Consult *Welch*—General Considerations Concerning the Biology of Bacteria, Infection and Immunity. Text-Book of the Theory and Practice of Medicine by American Teachers, edited by Wm. Pepper, M. D., LL. D., 1894, Vol. ?.

